# The Northern Lakes, a Summer Resort for Invalids of the South

**Published:** 1842-12

**Authors:** Daniel Drake

**Affiliations:** Professor in the Medical Institute of Louisville


### WESTERN JOURNAL

OF

### MEDICINE AND SURGERY.

---

DECEMBER, 1842.

---

ART. I.—*The Northern Lakes, a Summer Resort for Invalids of the South.* By DANIEL DRAKE, M. D., Professor in the Medical Institute of Louisville.

Much having been written on the comparative fitness of different places in the South, as winter residences for the invalids of the North, we propose to say something for the benefit of the valetudinarians of the former. Their present places of resort are chiefly the following—1. The Virginia Springs, where they *can* drink mineral waters of different kinds, and *must* breathe a mountain air which, although not in a high latitude, is invigorating. 2. The New York Springs, in a higher latitude, but on a lower level. 3. The Harrodsburg Springs of Kentucky, where art has contributed munificently to the comfort of invalids and the amusement of the gay, but the advantages of high latitude and alpine scenery cannot be enjoyed. 4. Long-Branch, Newport, Nahant, and other marine watering and bathing places; to which may be added, 5. A trip to the Falls of of Niagara, and 6. A voyage on the St. Lawrence to Montreal and Quebec. It is not our intention to declaim against any of these resources, but to add another, which we cannot but regard as superior to either, and, in reference to certain invalids and fashionable *ennuyés*, preferable to the whole. Before proceeding, however, let us inquire, what is necessary to give effect to a sojourn in the north by the classes of persons whose tastes or infirmities demand it. In the first place, as the majority are citizens, they should, in travelling for health or recreation, seek for places and scenes that will be in contrast with their homes. The invalid cannot recover, nor the fashionable rusticate, in a crowd. 2d. The places now frequented are not so far north as to give many of the advantages of a cool climate. 3d. They offer but few novelties. 4th. They do not abound in historical associations. 5th. Although the springs of New York, Virginia and Kentucky, are valuable in several forms of chronic disease, they are useless in others and injurious in some—while the invalid seldom proceeds to drink of their waters understandingly. 6th. The amusements and dissipations in which they abound, often tempt the infirm into unhealthy indulgences. 7th. It is, we believe, an admitted truth, that, in general, but a *part* of the benefit which results from visiting mineral springs, comes from the use of their waters.

We shall now proceed to illustrate the advantages offered in the hot season, by our northern lakes; in doing which we shall draw, chiefly, upon our own notes, made on a voyage of two months, for medical observation, during the past summer.

An inspection of a map of North America will show, that this numerous and extended group lie to the north of nearly all the States; their centre of gravity, the island of Mackinac, being in the meridian which separates Ohio from Indiana, cuts through the middle of Kentucky and Tennessee, and, dividing

Georgia from Alabama, reaches the Gulf of Mexico between East and West Florida. The eye still being kept on the map, the inhabitants of the South may trace out the routes by which they can reach and embark upon the lakes. First, they who reside west of this meridian, should ascend the Mississippi and Illinois, to Chicago, on the western shore of Lake Michigan; or taking the course of the Ohio river, cross the State of Ohio by land from Cincinnati to Sandusky City, or by the canal from Portsmouth to Cleveland. Second, they who reside east of the central meridian, may cross the mountains to Pittsburgh, and make their way, either by land or water, to the town of Erie in Pennsylvania; or take the New York route to Buffalo, at the eastern end of Lake Erie, or that to Oswego on Lake Ontario.

The eastern extremity of Ontario, of which Oswego is the "seaport," lies about one degree east of Washington City—Milwalke on Lake Michigan, and Navarino at the head of Green Bay, eleven degrees west; thus the voyage of the lakes extends through twelve degrees of longitude. Sandusky City, the most northern port of Lake Erie, is in latitude about 41° 35′; Gros Cap, at the entrance into Lake Superior, in lat. 45° 29', giving a range between these parallels of four degrees. Such are the present limits, but when steamboats shall be placed on Lake Superior, which must be done at no distant time, the voyage will be extended many degrees to the west, and north to the 49th. As it is, the people of the South, from Louisiana to Carolina, may journey and sojourn, during the heats of summer, in a climate from 10° to 15° north of their own. The central lakes, Erie, Huron, and Michigan, either of which is more extensive than all the lakes of Europe taken together, are at present, and, indeed, will continue to be, the chief places of resort.

The voyage from Buffalo to Chicago is more than 1200 miles; on which the traveller is carried by long stretches to the west, the north and the south; never out of sight of land on Erie, and not long on Huron and Michigan. During the three summer months, he will seldom encounter heavy gales; but from the shallowness of Lake Erie, it becomes agitated by gentler winds, and it is not uncommon for the invalid to experience the unwelcome benefit of a turn of "seasickness." When an occasional tempest stirs up the deeper waters of Huron or Michigan, a more formidable agitation, such as the Atlantic might not disown, arises, but this is of rare occurrence. Hence the dangers of a voyage upon the lakes are almost limited to fire, as sawyers do not exist, and rocks are scarce, while the ample "sea room" is an adequate guaranty against the fatal rencontre of boat with boat, which, on our rivers, destroys or cripples so great a number. From Buffalo to Chicago and Milwalke, there is a daily line line of steam packets, not inferior in size, strength and convenience to any in the United States; from Buffalo to Navarino, at the farther end of Green Bay, a regular packet runs every fortnight, and from the same eastern port to the *Sault St. Marie,** 15 miles from Lake Superior, one or two boats go every summer. The whole of them touch at the island of Mackinac both going and returning. In addition to these, there are many small steamers running between all the intermediate ports of Lake Erie, Lake St. Clair and the beautful river St. Clair, up to Fort Gratiot and Port Huron in the State of Michigan, and Port Sarnia, in West Canada, all at the lower end of Lake Huron. Some of these boats also ascend the Sandusky and Maumee rivers to their Rapids, at Lower Sandusky on the former, and the towns of Maumee and Perrysburgh on the latter. From Sandusky City, from Toledo on the Maumee Bay, from Monro on the River Raisin, and from Detroit, rail road cars run from 30 to 80 miles into the interior of Ohio and Michigan; finally, at Mackinac and the *Sault* (Rapids of the St. Mary), there are fur traders' skiffs, and birch canoes, with which French *voyageurs* and *Indians* are ready, at all times, to carry travellers along or across the numerous straits which there connect together the three greatest lakes of the continent. Thus, facilities for visiting every interesting locality, in and around these Mediterranean seas, are entirely within the reach of those who may embark upon their bosom.

---

* Throughout the north called—*The Soo.*

But the means of acquiring a knowledge of their area, depth, temperature, color, and other characteristics, may not always be available to those whom we hope to turn upon them; and we shall, therefore, devote a page or two to those subjects; for travel, to be beneficial, should excite the mind, as well as agitate the body.

The topographical engineers of the United States, and of New York, Ohio and Michigan, and the Historical Society of the last, have published much interesting information on these topics, which we shall use in connexion with our own limited observations.

The estimated area of Lake Ontario is 6,300 square miles of Erie 9,600—of St. Clair 360—of Huron 20,400—of Michigan, including Green Bay, 24,400—of Superior 32,000, making an aggregate of more than 93,000 square miles. From Ontario to Superior, there is a rise, which, however, is by unequal steps. The surface of the former is 232 feet above the tide water of the St. Lawrence; Erie is 333 feet higher, or 565 feet; St. Clair 6, or 571; Huron and Michigan 13, or 584; and Superior, 44 feet higher still, or 628 feet above the level of the ocean.

Of the whole, the little St. Clair is the shallowest, not averaging more than 20 feet; Erie, varying much, has no great depth any where, and does not average more than 84; Ontario has a mean depth of 500; Superior of 900, and Michigan and Huron of 1000. The deepest soundings of the whole are in the last—off the mouth of Saganaw Bay, traversed in the voyage to Mackinac, where the lead has sunk 1800 feet, more than 1200 below the level of the Atlantic Ocean, without reaching bottom.

The temperature of the lakes is diminished by latitude and depth. Thus, in reference to the former, we found a difference of 10 or 12 degrees, between the shoal water on the south side of Lake Erie and the north of Huron; and in reference to the latter, we observed, in crossing Lake Michigan from Green Bay to Mackinac, that the thermometer first sunk from 76° to 58°; then, after passing the centre of the lake, and entering the straits of Mackinac, gradually rose to 64°. Again, on leaving the harbor of Mackinac, several days afterwards, it was 61°; in the deep waters of Lake Huron it sunk to 53°, while at the lower end of the Lake, in shallow water, it rose to 65°. We made but a single observation on the heat of the water *below* compared with that of the *surface* of the *same* spot. It was in Huron near the Island of Mackinac, where we found the surface water, and that 200 feet deep, of the same temperature—56°. Travellers, then, who cross the lakes, and wish for cool water, need not draw it from their depths, as that of the surface, at any given point, would be of the same temperature with that beneath. This uniformity results, no doubt, from the transparency of the upper lakes, by which the rays of the sun penetrate them, without meeting with solid matters in suspension to elicit their heat; it is like their passage through the cloudless atmosphere, which they do not warm.

The shallow lakes, Erie and St. Clair, are less transparent than the deeper ones above; for the winds agitate them to the bottom, which throws up a portion of its slime. The clearness of the deeper is so crystalline, that the Indians spear their fish at the depth of 30 feet; and when the voyager looks from his boat into a still greater depth, he sees the pebbly bottom, with a distinctness which deceives him into the belief that it is but a feet below him.

The color of the lakes varies with their depth and transparency. Where shallow and somewhat turbid, it is of a feeble green, inclining to a light drab; where of greater depth, and perfectly transparent, the green is much deeper, and runs into an azure teint.

The lakes have no lunar tides, but are subject to rises and fluctuations, depending on other causes. From 1819 to 1838 they gradually rose five feet three inches; after which, they fell much more rapidly than they had risen. By this rise, fruit and forest trees, some of the latter ascertained to have been more than one hundred years old, were destroyed; and consequently there had not been an equal rise within that period. This gradual and remarkable swell, is attributed by the geologists to extraordinary rains and melting snows. There is, also, a semi-annual rise and fall of less extent, depending on the supplies which the rains and dissolving snows of spring and summer furnish; and on the congelation, in winter, of all the water which falls, with much of that already in the rivers of the north. The winds impart a daily agitation, which gives a rise and fall along all the shores, preventing stagnation and rendering the marshes which border them, less unhealthy than they otherwise would be. Lastly, the same cause, acting for some time in one direction, will pile up the waters, whence they return, when it is perfectly calm, in what are called ground swells, to overflow the shores from which they had been driven.

Some remarks on the summer climates of the lakes naturally follow those which have just been made.

When the south-west winds, which have traversed the vast plain separating the Gulf of Mexico from the lakes, reach the shores of the latter, they are necessarily dry and hot. Hence the temperature of Buffalo, Erie, Cleveland, Sandusky, Toledo, Detroit and Chicago, in the average latitude of 42°, is quite as great as their position should experience—greater, perhaps, than the traveller from Louisiana or Carolina would expect. But the duration of these winds is at no time very long, and whenever they change to any point of the compass N. of W., they bring down a fresh and cool atmosphere, to revive the constitutions of all whom they had wilted down. These breathings from the north, descend from the highlands around Lake Superior, which are nearly as elevated as the mountains of Pennsylvania and stretch off beyond the sources of the Mississippi to the Chippewyan or Rocky Mountains. In passing over that lake, with Michigan and Huron immediately south of it, the temperature of which in summer, as we have already seen, is less than 60°, these winds suffer no increase of heat, but become so charged with moisture from the extended watery surface, as to exert on the feelings of the people along the southern shores of Erie and Michigan, a most refreshing influence.

From the hour that the voyager enters Lake Huron at the head of St. Clair River, or Michigan at Chicago, he ceases, however, to feel the *need* of such breezes from the north-west; for the latitude which he has then attained, in connexion with the great extent of the deep waters, secures to him an invigorating atmosphere, even while summer rages with a withering energy in the south. The axis of each of these lakes is nearly in the meridian, and every turn made by the wheels of his boat, carries him further into the temperate and genial climate of the upper lakes. Entering it by either of the portals just mentioned, he soon passes the latitude of 44°, and has then escaped from the region of miasms, musquitoes, congestive fevers, calomel, intermittents, ague cakes, liver diseases, jaundice, cholera morbus, dyspepsia, blue devils and duns!—on the whole of which he looks back with gay indifference, if not a feeling of good-natured contempt.

Every where on the shores of the lakes, from Ontario to Superior, if the general atmosphere be calm and clear, there is, in summer, a refreshing lake and land breeze; the former commencing in the forenoon, and continuing, with a capricious temper, through most of the day; the latter setting in at night, after the radiation from the ground has reduced its heat below that of the water. These breezes are highly acceptable to the voyager while in the lower lake region, and by no means to be despised after he reaches the upper.

But the summer climate of the lakes, is not the only source of benefit to invalids; for the agitation imparted by the boat, on voyages of several days duration, through waters which are never stagnant, and sometimes rolling, will be found among the most efficient means of restoring health, in many chronic diseases, especially those of a nervous character, such as hysteria and hypochondraism.

Another source of benefit, is the excitement imparted by the voyage to the faculty of observation. At a watering place all the features of the surrounding scenery are soon familiarized to the eye, which then merely wanders over the commingled throngs of valetudinarians, doctors, dancers, idlers, gamblers, coquettes and dandies, whence it soon returns, to inspect the infirmities or *tedium vitæ* of its possessor; but on protracted voyages, through new and fresh regions, curiosity is stirred up to the highest pitch, and pleasantly gratified by the hourly unfolding of fresh aspects of nature; some new blending of land and lake—a group of islands different from the last—aquatic fields of wild rice and lilies—a rainbow walking on the "face of the deep"—a water spout, or a shifting series of painted clouds seen in the kaliedoscope of heaven.

But the North has attractions of a different kind, which should draw into its summer bosom those who seek health and recreation in travel. From Ontario to Michigan, the voyager passes in the midst of spots consecrated to the heart of every American; and deeply interesting to all who delight to study the history of their native land. The shores and waters of the lakes, so often reddened with the blood of those who fought and died in the cause of their country, will present to the traveller of warm and patriotic feelings, scenes which he cannot behold without an emotion under which real diseases may abate, and the imaginary be forgotten.

Should he enter the lakes at Chicago on Lake Michigan, he finds himself on the spot where the fort, bearing that name, at the outbreak of the last war with England, was surrendered to the Pottawatomie Indians, who sacked it; and then pursuing, overtook and nearly annihilated the little command of Capt. Heald—the lamented Indian spy, Capt. Wells, being among the slain.

Or should he first visit Ontario instead of Michigan, he will find that Oswego, the town of his embarkation, was once assaulted and taken; turning eastwardly, he will pass near the spot where Major Appling performed an achievement for which he was promoted; and reaching Sackett's Harbor, he may renew his admiration for the military talents of Brown, first displayed in the preservation of that place. He can then cross the lake to York, and survey the scene of one of his country's most brilliant victories, saddened, however, by the death of the accomplished and courageous Pike, cut off in the bloom of life. Advancing up the lake to Niagara, or arriving on that celebrated river by any other route, he is at once in the midst of battle fields, where the voice of the mighty cataract was no longer heard above the din of war. Visiting the site of the British Fort George, his heart will palpitate at the recollection of the irresistible energy with which Scott and Boyd and Hindman, and their associates and men, effected its capture. Crossing to Fort Niagara, he will in imagination hear the bombardment from sunrise till dark, which threatened a destruction, that was only averted by the indefatigable perseverance and skill of Major Armistead.

Ascending the river, he will survey on the Canada shore, the rocky heights of Queenstown, stormed and taken, retaken, then lost, recovered again, but finally retained by the British, with the capture of the remnant of as brave an army as ever drew sword; he will admire anew the invincible courage of Col. Van Ranselaer the commander, of Scott and Fenwick, of Wool and Christie, of Totten and many others; he will look back, with renewed contempt, upon the twelve hundred militia, who stood as idle spectators on the American shore, refusing to cross and relieve their brethren; finally, he will not withhold a passing tribute of respect to the memory of Gen. Brock, the bravest enemy who fell on that eventful and trying day.

Rising upon the upper level of the Niagara, he will soon reach the spot where an invasion of Canada was projected, undertaken, suspended, renewed, but at last, with an ardent and willing army at his command, ingloriously abandoned by Gen. Smyth. But other and more cheering associations will rise around him when he crosses into Canada. At the sight of Fort Erie, Chippewa, and Lundy's Lane, he will recall deeds of valor which gave a new aspect to our national character; the matchless gallantry of Brown, of Ripley, of Miller, of Scott, of Jessup, of Leavenworth, of Trimble, with a long and brilliant catalogue of kindred names, will rush upon his memory; and the unconquerable courage of our soldiery, when properly disciplined, fill him with a renewed admiration of his own people and his own land.

Having listened to the deep and deathless voice of the gathered waters as they plunge below, and lingered on battle fields consecrated to his country's glory, till his soul is filled with emotions of the sublime in the natural and moral world, and images of grandeur play around him, like the sparkling bows which hover over the cataract, he may depart on his voyage, and will feel that the demons which infested his shattered nerves have been already cast out.

Embarking at Buffalo for the West, he should limit his first voyage to Erie, in Pennsylvania. Here he will find on the margin of the lofty parapet which supports the town, the ruins of the old fort *Presque Isle;* the key by which the French, from Canada, passed into the valley of the Alleghany, when they erected *Fort Du Quesne,* where Pittsburgh now stands. Advancing a few hundred yards to the east, on the margin of the same plain, he may pluck a straggling sweet briar from the spot where, for many years, reposed the bones of the gallant and restless Wayne, interred on his return from Detroit in 1796. Close at hand he will see and may enter a block-house, erected in 1812, and through its port holes look out upon the winding and shallow channel, through which the enterprising Perry contrived to convey his dismantled fleet, in despite of the vigilance of Com. Barclay, who commanded the Lake. Finally, by casting his eye to the peninsula beyond, he will see rising out of the shallow waters, the sunken hull of one of the ships which soon afterwards brought down the flag of that officer, and compelled the surrender of his fleet.

Re-embarking, he will next stop at the beautiful city of Cleveland, whence he may cast his eye a few miles up the lake, and contemplate the iron bound coast on which, during a storm, the fleet of Gen. Bradstreet, carrying three thousand men, came nigh being entirely shipwrecked. As this was in 1763, it is a part of our own history. He was then on his way to relieve Detroit, for twelve months besieged by Pontiac, the most distinguished of all our Indian warriors.

Landing at Sandusky City, he will be on the spot where Bradstreet on this voyage dispersed a community of Indians and destroyed their fields and habitations. He may here take a smaller boat, and ascend Sandusky Bay, on a voyage of 30 miles, in which, if he choose, he may stop and explore the plaster quarries of the western shore, where he will detect nature in the very act of changing secondary limestone into gypsum. But continuing his voyage, in a narrow and winding channel, through the grassy bay, which at each breath of the steamer sends up flocks of white cranes and other water fowl, he finds himself in a few hours at the town of Lower Sandusky, on the eastern edge of the celebrated black swamp. Here he stands upon the spot where the young and fearless Croghan planted the single gun, which filled the ditches of Fort Stephenson with the dead bodies of its invaders, and led to the precipitate retreat of an overwhelming British and Indian force.

Should the traveller then take the stage, on a pleasant road along the river, he will scarcely leave the town, before he passes among trees scarred with bullets in the severe contest between Maj. Ball, commanding a small squadron of horse, and an ambuscade of an equal number of Indians, in which the latter were all killed. Proceeding on his journey, he will, before night, reach the village of Upper Sandusky, for a long time the residence of the respectable tribe of Wyandot Indians, now about to emigrate to the distant west. When within three miles of this spot, he will find himself on the ill-fated battle ground of Col. Crawford, defeated and captured in 1782. Turning thence to the Tyamochtee, at the distance of a few miles, he will see upon its banks an old apple tree, indicating the site of the Delaware town, commanded by Capt, Pipe; but will in vain seek for the *precise* spot where Crawford perished at the stake, pierced from the crown of his head to the sole of his foot with firebrands.

On his return he may take the stage, and dine in Tiffin on the banks of the river, whence a railroad car, in three hours, will restore him to Sandusky City.

He may now embark for Maumee Bay, and landing in Toledo, will find, at the mouth of Swan Creek, the spot where the Indians, when they ascended the Bay to meet Gen. Wayne, left their women and children. In another hour he can reach Maumee City, at the head of the Bay and foot of the rapids; where the first memorial of the past, which meets his eye, will be the parapets and ditches of old Fort Miami, built by the British during the Revolutionary war, and held by them after the peace of 1783. Passing up the river bank about three miles, to a spot called by the French *Presqu' Isle,* he reaches the battle ground of Wayne; and by returning along the canal a mile from the river, passes through the very woods in which the army, under the command and animated by the spirit of that impetuous veteran, on the 22d of August, 1794, drove the Indians, at the point of the bayonet, back to their wives and children at the mouth of Swan Creek. The traveller once more standing beside the ramparts of Fort Miami, will recall the daring survey made by the victorious general and his *aids de camp*, under the guns of the fort, and the spirited correspondence which followed. Turning from the contemplation of a battle so decisive, that it led to the treaty of Greenville, and to a peace with the Indians of sixteen years' duration, he will see on the opposite side of the river, adjoining Perrysburg, the earthy foundations of Fort Meigs, so vigorously besieged by Proctor and Tecumseh, and so nobly defended by our beloved Harrison; and looking down at his feet, will discover that they rest upon the batteries from which the British bombarded the fort; and then will come up the harrowing recollection, that after the cannon of those batteries were spiked, the rash and fearless sons of Kentucky, under Col. Dudley, instead of crossing to the Fort, allowed themselves to be drawn into the woods by the Indians, and slaughtered beneath the very trees, under which twenty years before the victorious army of Wayne had reposed.

Again prosecuting his voyage, the traveller in a few hours may land at the mouth of the River Raisin, and by stepping into a railroad car, be put down in a few minutes nearly opposite the straggling but celebrated village of Frenchtown. The passage of a bridge and a walk of ten minutes, will bring him to the battle ground, where he will find guides who took part in the contest, and know every spot consecrated to the memory of some of the bravest men who ever fell in battle, or were barbarously murdered after being wounded and received as prisoners. The house on the opposite side of the river, where the Commander, Gen. Winchester, lodged apart from his heroic, but doomed little army, the night of the attack; the place where the gallant Major Madison stood, when he refused to obey the orders of his commander to surrender to the enemy; the grave of the fierce and fiery Capt. Hart, dug by the tempest when it uprooted a giant oak; lastly, the ashes of the house in which the dead and dying prisoners were consumed.

Hastening from this field of blood, the traveller re-embarks for Detroit. But if he should have taken a vessel at Sandusky, destined for that port, he will have passed between the Three Sisters, through the very waters in which the gallant Perry met the enemy and made him ours. Entering Detroit river, he finds on either hand, localities which alternately revive emotions of pride and mortification. On his right he sees Malden, at the opening of the war in the power of our army, but stupidly abandoned; on the left, Maguaga and Brownstown, where two detachments of our troops had severe, but unprofitable conflicts with superior detachments of British and Indians from Malden. In a few hours he reaches the ancient city of Detroit, and from its wharves surveys the opposite village of Sandwich, the landing place of Hull, when he invaded Canada. On entering the town, he is conducted to a tavern kept in the very house which was the head-quarters of that inglorious commander; while at the distance of a few squares, he sees magnificent edifices erected on the bastions of the fort which, regardless of the indignant remonstrances of a gallant army, he surrendered to Gen. Brock.

To relieve himself from the mortifying emotions which this spot will raise in his heart, the traveller, if he choose to make a short excursion into Canada, may take the track of Proctor's army, as he fled from Malden after Perry's decisive victory, and, standing on the banks of the Thames, contemplate the patriotism of the venerable Shelby, the heroism of Johnson, the military genius and unostentatious courage of Harrison the commander-in-chief, the bravery of all below him, the flight of Proctor and the death of Tecumseh—second only to Pontiac in talents and a dauntless devotion to the country and interests of the Red Man.

Returning to Detroit, the traveller may indulge in new reminiscences. He can go back a hundred and forty years, when its settlement was commenced by one hundred Canadian French from Montreal under Cadillac, and trace the struggles of its infancy with the surrounding hostile tribes; then think of its transfer to England in 1760, after the downfall of Montreal; of its siege by Pontiac in 1763, and of his original and cunning stratagem for its capture—frustrated only by the gratitude of an Indian woman, which prompted her to disclose the scheme to Major Gladwin, the commander; of the surprising perseverance and resources of Pontiac during the siege, which lasted for many months; of the arrival, at length, of General Bradstreet, with a numerous army; and of the retirement of Pontiac to the Illinois river, where he was soon after assassinated by an Indian. But the traveller's recollections will not be exhausted by these events; he will remember, that from the commencement of our revolutionary war, till the Indian treaty of Greenville, a period of 20 years, Detroit was the seat of that British influence which instigated and encouraged the Indians to make war upon the scattered settlements of the Ohio river, from its sources to its mouth; that Col. Byrd with six pieces of artillery actually penetrated into the heart of Kentucky; and that the ablest of her many heroic defenders, Gen. George Rogers Clarke, actually captured Hamilton, the Governor of Detroit, at Vincennes on the banks of the Wabash.

Leaving this ancient capital of the lakes, the voyager embarks for the Island of Mackinac, and standing on its summit, thinks over the eventful past. In front, reposing on a rock, are the battlements of Fort Mackinac; to his right, the shores on which the British landed, and at the dawn of day demanded and obtained a surrender of the fort, garrisoned only by a handful of men, ignorant of the declaration of war; underneath and around his feet he will see the foundations of another fortress, built by the conquerors; descending to the west, in a mile he arrives at the field where Col. Croghan, after effecting a debarkation for the purpose of reconquering the island, was repulsed with the loss of the brave and accomplished Major Holmes.

Returning to the village of Mackinac, he finds, in his way, a projecting rock, once the roof of an alcove or cavern, which, his guide will tell him, is called the Cave of Sculls, and then his memory will turn upon remoter events. As he emerges from the woods, and casts his eye across the strait, to the main land of Michigan, he will see, dim in the distance, the site of old Fort Mackinac, besieged, and taken by stratagem in the Pontiac war of 1763; he will recollect the story of its massacre; the thrilling incidents of Henry's captivity, and his concealment in the Cave of Sculls, under the vigilance of a generous and faithful Indian, who had adopted him as a brother.

Such are some of the historical associations connected with a voyage upon the lakes; and where else in the Union can the invalid and the patriot roam, to find localities so opulent in varied and affecting recollections—so accessible—so arranged upon the thread of travel! We may fearlessly affirm, that in this respect, the lakes of the north take precedence over any other region of our beloved country. Their deeply wooded shores yield a bountiful harvest of facts to the historian; while their green waters reflect images of glory, sadness and shame, which the poet and orator will embody and bequeath to posterity.

Exciting and curative as it must be, for the valetudinarian of taste and intelligence, to pass a summer amidst scenes which can thus touch his feelings, and beguile him into forgetfulness of his sufferings, whether real or imaginary, he has still other sources of profitable recreation, in the voyage we are gratuitously prescribing; and in pointing them out, we shall designate the places on the lakes, at which he should repose and look around him.

The towns of Buffalo, Erie, Painesville, Cleveland, Sandusky, Manhattan, Toledo, Maumee, Perrysburg, and Monro, afford good accommodations and excellent society, but as we cannot recommend a *protracted* sojourn on the shores of Lake Erie, in the heat of Summer, we shall leave them, to speak of places farther north.

Detroit, lying above the latitude of 42°, offers to the people of the South a salutary change of climate, as well as a place of elegant repose after the fatigue and excitement of a long journey; and being the key to the upper lakes, is a suitable spot on which to fit out for more distant points. Its society, in cultivation and hospitality, is among the best in the Union. It is the permanent residence of the urbane old soldier, Brigadier General Brady—a lieutenant in Wayne's victory, a wounded colonel in the bloody battle of Niagara, and now the commander of our northern posts; also, of the Stuarts, uncle and nephew, who figure so largely in Washington Irving's Astoria—the latter, at present the General Superintendent of Indian Affairs for the north; of H. R. Schoolcraft, extensively known as a writer on the Indians; of Dr. Douglas Houghton, the indefatigable Geologist of Michigan; and of Col. Henry Whiting, who has dignified his profession by the cultivation of letters. In addition to these, the city is the residence of the Governor and officers of Michigan; of the Federal District Judge; of the Episcopal and Roman Bishops of the State; and of many highly intelligent gentlemen, who have been attached to the army, and the Indian Department, or engaged in the Indian trade. Finally, as she resides in the neighborhood, the traveller may have the good fortune to meet with Mrs. Clavers, whose vivid pictures of life in Michigan, foreshow that she is destined to become one of our most popular writers.

Detroit, once a French village, is now so thoroughly Americanized, that it is chiefly among the *names* of its inhabitants, we find the lingering evidences of its origin and early condition; but above and below the city, on either bank of the river, the habitations and wind-mills of the Canadian French, constitute a striking feature of the landscape, and contribute to that variety, which imparts efficacy to travel, undertaken either for health or recreation.

Of the two railroads from Detroit, one may be travelled 18 miles—the other 80. The latter runs nearly west, and makes the first link in the route to Lake Michigan. Thus, the invalid traveller, in a few hours, may place himself in the interior of the State, and wander among its miniature lakes. Stopping in Ann Arbor on his return, he may visit the new State University, with its magnificent museum of the natural productions of Michigan, from Erie to the distant shoresof Lake Superior, arranged by the State Geologist.

The town of Port Huron, at the lower end of the lake bearing that name, may be advantageously visited for two or three days. It is built on a low *dune*, and through its centre flows Black River, whose waters have the color of diluted ink. A mile above, he can visit and inspect Fort Gratiot. On the opposite side of the strait, he may spend a day, in the vicinity of Port Sarnia, among a permanent band of Chippewa Indians, rising into civilization under the efforts of a Methodist missionary, aided by the Canadian government.

Chicago and Milwalke, on the western shore of Lake Michigan, in lat. 42°–3°, although, like the towns of Lake Erie, too far south for the summer *residence* of travellers, present the phenomena of a refined society, and elegant towns built, like the ancient Tadmor, in the wilderness; but the facilities for reaching them are very different, as a daily line of commodious steamers communicates with both; and affords an opportunity for those extended voyages, which give to the lakes of the north a part of their pre-eminence over all other places of resort on the continent.

Green Bay, the name of a beautiful gulf of Lake Michigan, is also the name of a township at its head, within which are Fort Howard, not now garrisoned, and the beautiful, young, but not growing villages of Navarino and Astor, adjoining each other. Between them and the fort, all built on low *dunes*, Fox River, having its origin in the little lake Winnebago, enters the Bay. Around its mouth, in all directions, are grassy marshes communicating with the Bay; but the traveller need not feel apprehension at leaving his boat and awaiting her return, in a fortnight; as the fevers of the south are almost unknown at this spot, the latitude of which is nearly 45°. The chief motives of sojourn here, are its distance to the north west, the wildness of the surrounding country, and the proximity of the Oneida and Menomonee Indians; the former, emigrants from New York, and considerably advanced in civilization—the latter, in a state of original barbarism. Families of both may be seen daily in the villages, where they bring their manufactures, of which the traveller may readily obtain many curious and amusing specimens. But the island scenery of Lake Michigan and Green Bay, is perhaps the greatest attraction to this distant voyage. It is not easy to conceive of any thing more beautiful. Green waters sending up green islands, relieved by white girdles of lime-rock interposed between the waves and trees, are objects which the most querulous dyspeptic could not behold with indifference; nor the traveller of taste, look upon without rapture.

The Sault, or Rapids of the St. Mary, is a still more interesting place of sojourn than the last. By a circuitous navigation, it is 90 miles above Mackinac and 15 below Lake Superior. The voyage, chiefly through straits winding among innumerable islands, is attended with some difficulties, while there is no emigration and not much travel in that direction, and hence but few steamboats go thither. Schooners, however, visit the *Sault*, and the Indians and Canadian French ply between it and Mackinac in skiffs and birch canoes. As we ascend the St. Mary, hills and ridges of primary rock, rise to such a height *compared to the shores below*, as to present something of mountain scenery.

As the *Sault* has the latitude of forty-six and a half degrees, it is the most northern summer residence which our country affords; and corresponds with Tampa Bay, as a place of resort in the winter. It was settled, as a Canadian fur trading establishment, nearly two centuries ago; and a long experience proves, that its summer climate is still one of the most healthy on the continent. It is still *but* a trading establishment, protected by Fort Brady, and inhabited by a mixed population of Americans, French and Indians. The British North West Company have a factory on the opposite shore of the river. An Episcopal Missionary keeps a school for Indian children on the same side, and there is another in the village supported by the Methodist Church. Wild and distant, then, as this place may seem to the fashionable valetudinarian, he will perceive, that on going thither he will not pass beyond the companionship of clergymen, teachers, officers, merchants and other members of civilized society; while every hour in the day will furnish him with groups of Chippewas; and every walk bring him to some one of their conical lodges, constructed of poles and flag mats, or cypress bark. He will also be in the midst of another class of men, almost as different from himself as the wildest Chippewas. The Canadian *voyageurs*, born and reared on the shores of the lakes, are a peculiar race, and add much to the picturesque aspect which society here presents. Equally animated and at ease, both on land and water, in the villages and the wilderness, among civilized men and the wildest Indians, they are the cosmopolites of the North, and the backwoodsmen of Paris; for whether encamped with Chippewas on the banks of the St. Mary, or engaged in transporting the travellers of the South to its source in Lake Superior, they display not a little of the gaiety of heart, and courtesy of manners, which characterize the French metropolis.

The canoe or skiff voyage up the St. Mary's, from the *Sault* to Lake Superior at *Gros Cap*, on the Canada side, is the most interesting of all the shorter excursions in the North. The traveller *may* go and return the same day, but he is too much hurried for accurate observation; and loses, moreover, the pleasure of encamping *a la Sauvage.* To enter a tent, or to *bivouac* on a sand bank, beneath pine trees, among grass and flowers, uninfested with gnats, musquitoes or snakes, and lodge for a night on a bed of fern, is a luxury of itself; but when we add the music of the waters at his feet, the solemn stillness of neighboring woods, the mingled merriment of the *voyageurs* and Chippewas, their clouds of tobacco smoke, and the draughts of hot tea made from the leaves of an adjoining bush, the hypochondriac rises in the morning from a delicious *midsummer-night's dream*, and goes on his way rejoicing.

In making this excursion, the disciple of good old Isaac Walton may watch the writhings of his worm in the deep and pellucid waters of the lake; the geologist break off specimens of wacke and old red sandstone from its banks; the *virtuoso* pick up shells and cornelians on the beach below; the botanist enrich his herbarium with flowers, the painter his portfolio with original sketches, and the lovers of nature at large, their imaginations with the wild and beautiful.

On returning, they may descend the *Sault* or Rapids, when, for nearly a mile, their little barque, as if by instinct, will rapidly pick its way through dashing currents and whirling eddies; while snatches of song by the Canadian boatmen, and the startling yells of the Chippewa Indians, will raise a chorus to the tumult of the waters, which their friends below as loudly echo back.

At the *Sault* resides Mrs. Johnson, the intelligent Indian mother-in-law of the two Schoolcrafts. The elder we have already mentioned; the younger, for seventeen years associated with the Chippewas, lives near her. This place is, also, the residence of John Tanner, captured more than fifty years ago, on the banks of the Ohio, in Boone County, Kentucky, and introduced to the reading public by Dr. James' Narrative. But a different inhabitant, of more interest than either to the dyspeptic and the gourmand, is the celebrated WHITE FISH, which deserves to be called by its classical name, *Coregonus albus*, which liberally translated signifies—food of the Nymphs. Its flesh, which is the cold and clear waters of the lake organganized and imbued with life, is liable but to this objection, that he who tastes it once, will thenceforth be unable to relish that of any other fish.

The Island of Mackinac is the last, and of the whole, the most important summer residence to which we can direct the attention of the infirm and the fashionable. True, it has no mineral springs; but living streams of pure water, cooled down to the temperature of 44°, gushing from its lime-rock precipices, and an atmosphere never sultry or malarious, supersede all necessity for nauseating solutioas of iron, sulphur and Epsom salts. An ague, contracted below, has been known to cease even before the patient had set his foot on the island, as a bad cold evaporates under the warm sun on a voyage to Cuba. Its rocky, though not infertile surface, presents but few decomposable matters, and its summer heats are never great enough to convert those few into miasms.

Situated in the western extremity of Huron, within view of the straits which connect that lake with Michigan, and almost in sight, if forest did not interpose, of the portals of Lake Superior, this celebrated island has long been, as it must continue to be, the capital of the upper lakes. The steamboats which visit the Rapids of the St. Mary and Green Bay, not less than the daily line from Buffalo to Milwalke and Chicago, are found in its harbor; and the time cannot be remote, when a small packet will ply regularly between it and the first. By these boats the luxuries of the South, brought fresh and succulent as when first gathered, are supplied every day. But the potatoes of the island, rivalling those of the banks of the Shannon, and the white fish and trout of the surrounding waters, yielding only to those of Lake Superior, render all foreign delicacies superfluous. We must caution the gourmand, however, against the excessive use of trout (*salmo amethystes*), which are said to produce drowsiness; for he who visits Mackinac, should sleep but little, lest some scene of interest may pass away unobserved.

The society of the town and post is every way equal to any reasonable expectation. There are three plain but comfortable houses of entertainment for strangers; and an increase in the latter would be followed by an immediate extension of accommodations, perhaps by the erection of a summer boarding house on the high eastern cliff of the island, which would be one of the most attractive in the United States. Several of the old French and mestizoes, are intelligent and courteous; there a number of traders and other gentlemen, whose long and extended intercourse with the natives, has qualified them to impart much curious and interesting information, and the sub-Indian agent, and the officers and chaplain of the army, are hospitable and gentlemanly; while last but not least, the female society is superior to what might be anticipated, in a town so remote as to connect savage and civilized men in their daily business.

The waters around the island are so narrow and tranquil, that many excursions of pleasure, in skiffs and canoes, may be performed with safety. Several other islands lie in view, and the coasts of the upper and lower peninsulas of Michigan, stretch off in four blue and beautiful curves. In a single hour Point St. Ignace may be reached, and in twice that time the traveller may stand on the site of old Fort Mackinac, already mentioned. These opposing shores constitute the portals of Lake Michigan. Passing through them, the enterprising traveller may perform a voyage on the coast of Michigan, to the Catholic, Ottowa villages of *L'Arbre Croche* and the *Petite Traverse;* and continuing it, reach the Chippewas of the *Grande Traverse* Bay, where he will find a Presbyterian Mission. At both settlements, he will see much of primitive Indian life, in the midst of a half civilized and grotesque population.

But should he *remain* on Mackinac, he will not be without interesting sights, for those which do not belong to it will come. The chief place of *Indian* trade for the north, and a stopping port for all who wander from lake to lake, it is the daily resort of Ottowas, Chippewas, and Pottawatomies, the two former, however, by far the most numerous. Here they bring their mokuks of maple sugar, their furs and dressed skins, their flag mats, embellished moccasins, feather-fans, and white birch boxes, baskets, and miniature canoes, ornamented with dyed porcupine quills. In addition to the few who are at all times visiting the island, a large number from the shores and islands of Lake Michigan and Green Bay, every summer congregate here, on their way to the Great Manitoulin Island, in the northern part of Lake Huron, to receive presents from the British Government for having fought against us in the war of 1812. A hundred conical lodges, on the beach in front of the village, with as many birch canoes drawn out of the water, may be seen at a single stroke of the eye, presenting with the fort above, the churches of the village below, and the steamboats and schooners of the harbor, a contrast of wonderful novelty and interest. But when they depart under a west wind, each having a square sail, and are seen from the eastern summit of the island, at every distance, the fleet of Lilliputian barques, might not inappropriately be likened to a scattered flock of gigantic swans, gracefully moving through the azure waters.

In conclusion, we must devote a page to the natural scenery of the island. Its entire circumference does not exceed ten miles. Seen as we approach from the east, it presents a mural precipice, of grey secondary limestone, rising 150 feet out of the green waters, and decorated on its brow with maples, oaks and evergreens. Over a chasm in the verge of this cliff, is a natural bridge, so narrow and elevated, that one of the exploits of the daring visiter, is to walk upon it. At a short distance in its rear, stands a conical rock, whose pinnacle overtops many of the forest trees, in the midst of which it has stood in solitary and undecaying dignity, while they, generation after generation, have mingled with the soil. On the western slopes of the island, there is an immense number of primitive boulders, from the granitic mountains beyond Lake Superior; lastly, on the very summit of the island, the naturalist may collect organic remains, and the curious peel white birch bark, on which, should they lack paper, they may write their notes, or correspond with their distant friends.

In a recess on the south-eastern side of the island, but a few feet above the surface of the lake, stands the grotesque village of Mackinac; where, side by side, are Canadian, cypress thatched cabins, and modern frames erected by our own people. On the cliff which overhangs it, sits Fort Mackinac, with its bristling cannon, and white-washed battlements. Half a mile in the rear is another plateau 75 feet higher, the site of old Fort Holmes, which we have already visited. From this summit, elevated far above all that surrounds it, the panorama is such as would justify the epithet to Mackinac, of Queen of the Isles. To the west, are the indented shores of the upper peninsula of Michigan; to the south, those of the lower, presenting in the interior, a distant and smoky line of elevated table land; up the straits, green islets may be seen peeping above the waters; directly in front of the harbor, Round Island forms a beautiful foreground; while the larger *Bois Blanc*, with its lighthouse, stretches off to the east; to the north are other islands at varying distances, which complete the archipelago.

When the observer directs his eye upon the waters more than the land, and the day is fair with moderate wind, he finds the surface as variable in its tints, as if clothed in a robe of changeable silk. Green and blue are the governing hues, but they flow into each other with such facility and frequency, that while still contemplating a particular spot, it seems, as if by magic, transformed into another.

But these mid-day beauties vanish before those of the setting sun, when the boundless horizon of lake and land seems girt around with a fiery girdle of clouds; and the brilliant drapery of the skies paints itself upon the face of the waters. Brief as they are beautiful, these evening glories, like spirits of the air, quickly pass away; and the grey mantle of night warns the beholder to depart for the village, while he may yet make his way along a narrow and rocky path, beset with tufts of prickly juniper. Having refreshed himself for an hour, he may stroll out upon the beach, and listen to the serenade of the waters. Wave after wave will break at his feet, over the white pebbles, and return as limpid as it came. Up the straits he will see the evening star dancing on the ruffled surface, and the lagging schooner flapping its loose sails in the fitful land breeze; while the milky way—DEATH'S PATH of the Red Man, will dimly appear in the waters before him. Behind, in the street, a lively group of Canadian French, of every shade of color between white and red, will gossip and shrug their shoulders; on one side, he will hear the uproar of a lodge of drunken Chippewas, with the screams of women and children, and the cackling of frightened hens; on the other will see, the sober and listless Ottowa, sitting in silent vacancy of thought, on the upturned keel of his birch canoe, his wife within the tent spreading cypress bark and flag mats upon the gravel, as lodgings for the night, while half a dozen children loll or play about the door, and as many half starved dogs curl up among them. Surrounded by such scenes, the traveller begins to realize that he is a stranger; when suddenly a new phenomenon appears and fixes the conviction. Every object becomes more visible, and, raising his eyes, he beholds the heavens illuminated with an aurora borealis, where he reads in fantastic characters of light, that he is, indeed, a sojourner in a strange land, and has wandered far from his friends and home in the sunny regions of the South.

Nov. 1842.

